# Theory of mind in chronic migraine with medication overuse assessed with the MASC

**DOI:** 10.1038/s41598-024-57559-0

**Published:** 2024-03-24

**Authors:** Sara Bottiroli, Alessia Rosi, Serena Lecce, Grazia Sances, Marta Allena, Roberto De Icco, Tomaso Vecchi, Cristina Tassorelli, Elena Cavallini

**Affiliations:** 1 Department of Brain and Behavioral Sciences, University of Pavia, Pavia, Italy; 2 Headache Science and Neurorehabilitation Centre, IRCCS C. Mondino Foundation, National Neurological Institute, Via Mondino 2, 27100 Pavia, Italy; 3 Applied Psychology Centre, IRCCS C. Mondino Foundation, Pavia, Italy

**Keywords:** Chronic migraine, Medication overuse headache, Mentalization, Ecological task, Psychology, Migraine

## Abstract

Theory of Mind (ToM) is the ability to infer one's own and others' mental states. Growing research indicates that ToM is impaired in Chronic Migraine with Medication Overuse (CM + MO). However, the research in this field has been conducted using static scenario-based tasks, often failing to test mentalization in everyday situations and measuring only performance accuracy. We filled this gap by administering the Movie for the Assessment of Social Cognition (MASC) to subjects with CM + MO compared to episodic migraine (EM). This test allows us to assess both affective and cognitive ToM and which, in addition to being accurate, also analyzes the type of error in attribution of mental states, distinguishing between hypo-mentalization and hyper-mentalization. Thirty patients suffering from CM + MO and 42 from EM were enrolled. Results showed that CM + MO patients were less accurate in mental state attribution than EM. In addition, compared to EM, CM + MO individuals were more impaired in the affective ToM dimensions and committed more errors of hypo-mentalization. In conclusion, the application of MASC in patients with CM + MO allowed for the detection of an alteration in their ability to correctly draw conclusions about other people's mental states. This latter contributes critically to appropriate social reactions and also, possibly, to satisfactory social interactions.

## Introduction

Migraine is known to represent one of the most disabling neurological disorders^1^. In most cases, attacks recur episodically (episodic migraine, EM), however in a small but relevant portion of patients, migraine acquires a chronic pattern (chronic migraine, CM) with at least 15 monthly headache days for at least 3 months, often associated with overuse of acute medications (MO)^2^. Current evidence suggests that, in addition to socio-demographic/lifestyle habits and medical history factors^3^, psychological aspects may also play a relevant role in the transformation of EM into CM, in particular in the case of MO. CM/CM + MO is indeed strongly associated with psychopathologies, including dependence, anxiety, depression, and personality disorders^4–10^, along with childhood trauma, life events, and alexithymia^11–13^. Importantly, comorbid psychopathologies bear a negative prognostic value in both the outcome of migraine and its response to treatments^4,14–16^. In addition to the well-known overlap between psychopathological comorbidities and CM + MO, scientific research interest is directed toward identifying all factors related to this complex clinical condition. According to the biopsychosocial model, there is a complex interrelationship between biological, psychological, and psychosocial vulnerabilities that mutually influence each other^17^. In other words, diversity in the expression of migraine, including severity, duration, and impact, results not only from the patient's biological characteristics, but also from the interaction with psychological state and social context, which can shape perceptions and response to the disease^18^.

Regarding social context, evidence suggests that migraine is a burdensome disease associated with many psychosocial difficulties—including social functioning^19^. Recent studies involving this clinical population have investigated those social cognitive abilities that underline social interactions, that is the mental operations that allow one to decipher information about the intentions and affective states of social interlocutors^20^. Among these social cognitive skills, Theory of Mind (ToM), the ability to infer one's own and others' mental states ^21^, is fundamental to guide social interactions as people’s mental states determine their actions^22^. ToM is characterized by two main aspects: cognitive and affective ^23,24^. Cognitive ToM is the ability to make inferences on others’ thoughts and beliefs, whereas affective ToM is the individual’s ability to make inferences on emotions and feelings.

As far as we know, ToM is a component of social cognition which is of concern in CM/CM + MO, as recent research in this area has shown mentalization deficits in this population. For instance, Bouteloup^25^, when comparing patients with severe EM and CM with healthy controls (HC), found difficulties in social and emotional cognition in the clinical populations. Raimo^26^ explored the neuropsychological correlates of ToM and found that CM patients had difficulties in the cognitive dimension involved in inferring the mental states of others. Romozzi^27^ compared CM + MO, EM, and HC with regard to the recognition of complex emotions, knowledge of one's own and others' mental states, and levels of alexithymia, and found impairment in all dimensions in CM + MO. Bottiroli et al. ^28^ have recently compared CM + MO patients to EM and HC in many aspects of social cognition, including abilities, beliefs, traits, and social relationships. What they found was that the two migraine groups performed similarly—and worse than HC—in terms of socio-cognitive abilities, but the CM + MO was more impaired in the affective dimension of ToM.

Two main considerations could be drawn from these investigations. Firstly, most ToM studies in this field have been conducted using static scenario-based tasks, such as stories grounded in false belief or faux pas understanding^25,26,28^. One or more characters are presented with limited contextual information and participants must infer the mental states of the character presented. Photographs of the ocular region of the face were also used^25–28^. Although these tasks are very useful in understanding ToM functioning, they often fail to truly test the ability to mentalize in a manner similar to that which occurs daily in real life. More specifically, these tasks lack ecological validity in that they require participants to use their ToM skills in static situations that are oversimplified, often unimodal (verbal or visual), based on few indicators or cues, and, finally, very different from real-life situations. Secondly, typical ToM tasks are able to capture only the presence or absence of mentalizing when evaluating right or wrong answers. However, beyond a complete lack of ToM, there are multiple ways in which mentalizing can go awry. Hence, other categories of mentalizing should be considered: less ToM (hypo-mentalizing) and excessive ToM (hyper-mentalizing). The relevance of this differentiation is supported by different patterns of difficulties which emerge in different clinical conditions^29–33^. For instance, autistic-spectrum and psychotic-spectrum conditions such as schizophrenia represent two diametrically opposite phenotypes of disorders of social cognition, which is underdeveloped in autistic-spectrum conditions and hyper-developed on the psychotic spectrum^34^. In particular, autism-spectrum disorder is characterized by poor ToM performance and impairments in the reasoning of intentions and emotions that highlight social conventions^30^. By contrast, ToM deficits increase in severity along the psychotic spectrum, with schizophrenia exhibiting impaired, inflexible, or extreme inferences regarding social cues and over-attribution of mental states and intentions^33^.

With all these assumptions in mind, the present study aimed to evaluate ToM in CM + MO compared to EM using the Movie for the Assessment of Social Cognition (MASC^35^), a task consisting of the presentation of a 15-min video clip of social interactions close to real life encounters in which participants are asked to identify and attribute mental states online. The reason for choosing to use this task is threefold. First, it allows an ecologically valid assessment of social interactions in everyday life, as it is a dynamic task that combines verbal and visual content. Second, this ecological task assesses two different aspects of ToM, namely affective ToM and cognitive ToM, thus assuring a more complete assessment. Cognitive ToM involves the representation of thoughts, intentions or beliefs, whereas affective ToM is the representation of feelings^23,36^. Third, in addition to accuracy scores, the MASC allows for the examination of the type of errors made in the misattribution of mental states to others, distinguishing between hypo-mentalization (under-attribution of intentions to others) and hyper-mentalization (over-attribution of intentions to others). According to our previous findings^28^, our hypothesis is that CM + MO patients are characterized by a marked impairment in the affective component of the MASC that differentiate them from EM. This hypothesis is also supported by previous evidence showing that affective ToM versus cognitive ToM may be particularly impaired in unipolar depression^37,38^, a condition that is particularly recurrent in CM + MO^39^. As error types, since no previous studies have been conducted on CM + MO, it is difficult to draw definitive hypothesis in the field of this clinical population. However, if we consider that patients with CM/CM + MO are typically characterized by alexithymia and difficulties in terms of emotional awareness^11–13^, it could be hypothesized that they are more prone to making errors characterized by reduced mental state attributions, i.e., hypo-mentalization. Similarly, patients with depression assessed with this same task were also found to be characterized by a tendency to hypo-mentalization^37^. Because this is the first study using MASC in CM + MO, we also administered a classic ToM task, widely used in previous research in this population^25–28^, the Reading the Mind in the Eyes (RMET^40^).

## Methods

### Participants

This is a cross-sectional case–control study conducted at the Headache Science and Neurorehabilitation Center (a tertiary referral center) of the C. Mondino Foundation in Pavia, Italy. We enrolled consecutive patients with stable EM (duration > 10 years) and patients with CM + MO for at least one year. An expert neurologist verified the eligibility criteria during the recruitment process based on history, headache diaries, and neurological evaluation (see below for inclusion and exclusion criteria). Participants completed a vocabulary test (drawn by the Primary Mental Abilities test)^41^ as a cognitive control variable of semantic knowledge. The study was approved by the Ethics Committee of San Matteo Hospital (Pavia, Italy) and written informed consent was obtained from all patients. The protocol followed the Strengthening the Reporting of Observational Studies in Epidemiology (STROBE) reporting guidelines for cross-sectional investigations^42^.

### CM + MO patients

Inclusion criteria were: (a) age > 18, < 65 years, (b) fulfillment of ICHD-III criteria for CM and for MO^2^, (c) onset of CM before 50 years of age. Exclusion criteria were: (a) dementia, (b) psychosis, and (c) mental retardation.

### EM patients

Inclusion criteria were: (a) age > 18, < 65 years, (b) fulfillment of ICHD-III criteria for migraine with or without aura^2^, (c) migraine duration ≥ 10 years, (d) no previous history of CM. Exclusion criteria were: (a) dementia, (b) psychosis, and (c) mental retardation.

### Procedure

Each consultation was performed by a headache expert that diagnosed the headache type and collected socio-demographic data and migraine characteristics. Participants also underwent an individual session of testing with a psychologist, lasting about 90 min, during which they completed ToM tasks and filled out self-report questionnaires, as reported below.

### Measures

#### ToM abilities

The MASC^35^ (Italian validated version^43^) is a video-based test assessing ToM ability in a comprehensive and more ecological way than traditional ToM tasks. It consists of a 15-min movie about four characters (two women and two men) meeting together to cook, dine, and play. Participants are required to answer 45 multiple-choice questions about the characters’ emotions, thoughts, and intentions. Of the 45 multiple-choice questions, 18 questions measure emotional and feelings states (i.e., affective ToM; e.g., “What is X feeling?”) and 27 questions measure thoughts and intentions (i.e., cognitive ToM; e.g., “What is X thinking/intending?”). Participants were provided with four response options: (1) an accurate ToM response, (2) an excessive-ToM response that refers to a hyper-mentalizing error, (3) a reduced-ToM response that refers to a hypo-mentalizing error, and (4) a no-ToM (no mentalizing) response. In addition, participants were provided with 6 control questions concerning non-mental details depicted in the video to account for memory and general comprehension abilities (e.g., “How was the weather on that evening?”).

Different scores were derived from this task. The MASC-Mental total score was calculated as the total number of correct responses to all ToM questions. Moreover, following the purpose of the present study, we calculated scores separately for the total number of correct responses for the affective items (MASC-Affective) and cognitive items (MASC-Cognitive). Furthermore, for each affective and cognitive item, we calculated, respectively, three error scores assessing inadequate mental state inferencing: (a) hyper-mentalizing; (b) hypo-mentalizing; (c) no mentalizing. Finally, we calculated the MASC-control score assessing non-social inferencing as the total number of correct responses to all control questions. All scores were transformed into percentages.

The RMET^40^ consists of 36 black-and-white photographs of the eye-region of the face, depicting a specific mental state. This test was employed to assess the ability to infer the affective mental states of other people (i.e. affective components of ToM). Participants were required to choose which one among four adjectives best described what the person in the photograph was feeling. The task also consists of a gender-recognition control test, in which participants were asked to judge the gender of the person in each of the 36 photographs.

#### Psychological and quality of life assessment

Levels of depression and anxiety were evaluated using the Hospital Anxiety and Depression Scale (HADS)^44^, whereas quality of life was assessed using the World Health Organization Quality of Life Brief Version (WHOQOL-BREF)^45^.

### Statistical analyses

Performance on the MASC was considered as the primary outcome. The sample size was calculated on this outcome, showing that a total of 64 participants was needed to discover a medium-sized effect having an f-squared of 0.15, with 0.80 statistical power and α = 0.05 in Multivariate analysis of variance (MANOVA).

Data are presented as means ± SD for continuous data and % for frequency data. One-way analysis of variance (ANOVA) and χ^2^ tests were conducted to compare groups on demographic variables. Multivariate analysis of variance (MANOVA) was performed on ToM measures with MASC and RMET scores as dependent variables and diagnosis (CM + MO vs. EM) as factor. When analyzing RMET and MASC scores, we took into consideration semantic knowledge (measured by a vocabulary test) as a cognitive control variable. This has been linked to improved performance in ToM abilities in previous studies ^46,47^. Additionally, we considered depression (measured by HADS-D) as an affective-control variable, as it has been associated with poorer ToM ability in prior research ^48^. We then examined group differences on all other variables with one-way analysis of variance (ANOVA).

### Ethics approval and consent to participate

The study was performed in accordance with the guidelines of the Declaration of Helsinki. Authors obtained local ethics committee (San Matteo Hospital, Pavia, Italy) approval of the protocol. All patients provided written informed consent in advance of study participation.

## Results

### Study population and demographic variables

Seventy-two subjects were enrolled, of which 30 suffered from CM + MO and 42 from EM. No significant differences between groups were observed in the demographic variables. Descriptive statistics about the sample are reported in Table 1.

**Table 1:** Demographic and clinical characteristics of patients enrolled in the study.

	CM + MO, n = 30 (mean ± SD)	EM, n = 42 (mean ± SD)
Age	43.67 ± 9.08	42.55 ± 9.94
Gender (female %)	83	84
Years of education	13.50 ± 3.31	14.67 ± 2.63
Vocabulary	46.27 ± 2.20	46.67 ± 2.53
Age at onset (years)	14.93 ± 9.80	16.78 ± 8.89
Duration of chronic headache (months)	71.31 ± 77.61	–
Days with headache per month	26.55 ± 4.45	6.55 ± 3.02
Average pain intensity (VAS)	7.36 ± 0.90	7.42 ± 0.70
MIDAS	70.25 ± 52.52	28.45 ± 10.03
HIT-6	65.62 ± 10.93	57.46 ± 9.84
Acute treatment		
NSAIDs	17%	
Triptans	25%	
Combination	49%	
Multiple drug classes	9%	
Prophylaxis		
None	10%	

*CM + MO* chronic migraine with medication overuse, *EM* episodic migraine, *vocabulary* range score 0–50, *VAS* Visual Analogue Scale (range 0–10), *MIDAS* Migraine Disability Assessment Scale (range score 0–270), *HIT-6* headache impact test (range score 36–78), *NSAIDs* nonsteroidal anti-inflammatory drugs.

### Psychological and Quality of Life assessment

Descriptive data and statistics for psychological and quality of life assessment are reported in Table 2. Regarding the HADS, significant group differences were found in the depression subscale, where the CM + MO group had significantly higher scores than the EM group, but not in the anxiety subscale. Concerning QoL, group differences emerged in the WHOQOL-BREF, where the CM + MO group had significantly lower scores compared to the EM group.

**Table 2:** Means and standard deviations of ToM tasks and psychological characteristics for each diagnostic groups.

	Range	CM + MO, n = 30 (mean ± SD)	EM, n = 42 (mean ± SD)	Statistic		
				*F*	*p*	*η*^*2*^_*p*_
**Movie for the Assessment of Social Cognition (MASC)**^a^ ***Accuracy scores***						
MASC—mental	0–100	59.70 ± 9.92	66.14 ± 8.89	5.94	**0.017**	0.080
MASC—control	0–100	81.67 ± 22.04	84.92 ± 5.53	0.06	0.800	0.001
MASC—affective	0–100	48.52 ± 12.20	57.27 ± 12.96	6.36	**0.014**	0.085
MASC—cognitive	0–100	67.16 ± 11.50	72.05 ± 9.89	2.37	0.128	0.034
***Error scores***						
MASC—affective hyper-mentalizing	0–100	14.26 ± 7.67	14.55 ± 11.77	0.19	0.662	0.003
MASC—affective hypo-mentalizing	0–100	28.37 ±8.56	20.37 ± 8.56	12.62	**0.001**	0.157
MASC—affective no-mentalizing	0–100	8.89 ± 7.66	7.67 ± 7.15	0.48	0.493	0.007
MASC—cognitive hyper-mentalizing	0–100	17.41 ± 8.97	14.64 ± 7.17	1.27	0.263	0.018
MASC—cognitive hypo-mentalizing	0–100	9.51 ± 7.94	7.16 ± 6.16	1.68	0.199	0.024
MASC—cognitive no-mentalizing	0–100	5.93 ± 5.11	5.55 ± 4.99	0.13	0.723	0.002
**Reading the Mind in the Eyes Test (RMET)**^a^						
RMET experimental	0–36	21.00 ± 3.46	23.81 ± 4.09	9.58	**0.003**	0.123
RMET control	0–36	35.50 ± 1.01	35.29 ± 0.86	0.43	0.514	0.006
**Hospital Anxiety and Depression Scale (HADS)**^b^						
HADS anxiety	0–21	7.50 ± 4.59	5.71 ± 4.33	0.57	0.452	0.008
HADS depression	0–21	7.03 ± 4.54	4.83 ± 3.83	4.94	**0.030**	0.066
**The World Health Organization Quality of Life (WHOQOL-BREF)**^b^	8–40	25.97 ± 5.23	29.46 ± 4.49	7.42	**0.009**	0.117

*CM + MO* chronic migraine with medication overuse, *EM *episodic migraine: ^a^df (1,68); ^b^df (1,70).

### ToM abilities

Descriptive and statistics for ToM measures are reported in Table 2. For the MASC Mental vs. Control items, no significant influence of diagnosis was observed (Wilks' *λ* = 0.92; *F*[2,67] = 2.96; *p* = 0.059, *η*^*2*^_*p*_ = 0.081). Regarding the MASC Cognitive vs. Affective items of the MASC, there was a significant influence of diagnosis (Wilks' *λ* = 0.91; *F*[2,67]  = 3.45; *p* = 0.037, *η*^*2*^_*p*_ = 0.093), where the CM + MO group reported significantly poorer performance on Affective items compared to the EM group, but not on Cognitive items. Finally, looking at MASC analysis of error responses (hyper-mentalizing vs. hypo-mentalizing vs. no mentalizing) in the Affective vs. Cognitive items, results showed a significant influence of diagnosis (Wilks' *λ* = 0.80; *F*[2,63]  = 2.59; *p* = 0.026, *η*^*2*^_*p*_ = 0.198). The CM + MO group made significantly more hypo-mentalizing errors in the Affective items compared to the EM group. We found no group differences in the other categories of errors.

Regarding the RMET Experimental vs. Control condition, there was a significant influence of diagnosis (Wilks' *λ* = 0.87; *F*[2,67] = 4.82; *p* = 0.011, *η*^*2*^_*p*_ = 0.126), where the CM + MO group displayed significantly poorer performance on the Experimental condition compared to the EM group, but not group differences in the Control condition. In all previous analyses, the covariates vocabulary and depression did not have any significant impact on RMET and MASC scores (*ps* > 0.099).

Moreover, given that the number of correct responses and of errors are count data, yet the error evaluation cannot be converted into counts which remain meaningful, we also adopted a non-parametric approach, considering a quantile regression on the median in order to check the correctness of the results, and found that said results did not change.

## Discussion

The main goal of the present study was to examine ToM in CM + MO patients compared to EM by using an ecologically valid and sensitive tool, that being the MASC^35,43^. This task offers a unique advantage for studying individual differences in ToM performance, assessing aspects of both cognitive and affective components of mentalization and providing an in-depth examination of correct and incorrect patterns of mentalization. Due to the video-based format which allows for a multimodal presentation of the task, MASC may be more ecologically valid^49^ than conventional ToM tasks or other purely verbal tasks.

Similarly to previous studies^25–28^, we confirm impaired ToM performances in CM + MO with respect to EM when using the RMET. This finding supports the existence of socio-cognitive deficits in this population characterized by chronic and recurrent pain, which may have diminished or impaired natural engagement with the mental and emotional states of others. The interesting and completely new aspect of the present study is that we extended and deepened this topic with a more ecological task, the MASC. Thanks to this instrument, we were able to not only identify differences in the mental condition between the two clinical groups but also to pinpoint the dimension (affective or cognitive) in which CM + MO patients were most impaired. In addition, we managed to gather information about the type of errors (hyper-mentalizing, hypo-mentalizing, or no mentalizing) made in misattributing mental states to others. Our findings revealed that individuals with CM + MO had more pronounced impairments in the affective ToM dimension than in the cognitive ToM dimension, when compared to subjects with EM. Tasks designed to assess affective ToM require decoding mental states from perceivable social information (e.g., tone of voice, body posture, or facial expression), while those designed to assess cognitive ToM require reasoning about mental states by integrating contextual and historical information about a person (e.g., idiosyncratic experiences, knowledge, attitudes) in order to understand behavior^50^. Hence, it could be argued that CM + MO is associated to specific difficulties in decoding processes. Two explanations can be advanced in order to comment on this result. First, these affective difficulties should be considered as being associated to alexithymic characteristics which usually feature in these patients. There is indeed a robust body of research showing that alexithymia is an important feature of CM/CM + MO, being reported in nearly 70% of patients with this diagnosis^51^. According to this interpretation, research in other areas showed an association between difficulty in identifying and describing feelings and responding atypically to emotional cues in others^52–54^. Second, this impairment could also be associated to the greater depressive symptomatology found in CM + MO patients, which may have made them less sensitive to depict others’ nonverbal information and emotional contents. As previously mentioned, there is indeed evidence^37^ demonstrating deficits in the affective dimension of the MASC in unipolar depressive patients, as well as existing associations between depression and deficits in ToM affective performance^55^. Our results did not fully support this interpretation, as depressive symptomatology did not explain diagnosis-related differences in ToM abilities, although CM + MO patients were characterized by more pronounced symptoms. However, we believe that the HADS did not allow us to fully depict the existing association between depression and affective impairment in ToM. Indeed, those^37^ who found such an association used preexisting depressive diagnoses and not the self-report questionnaires which we employed. Future research is therefore needed, using clinical instruments, to further explore the association between affective difficulties in ToM and depression (and also alexithymia), to determine whether this impairment in CM + MO is a stable, trait-like phenomenon, or whether this impairment is state-dependent.

Our results also highlighted that the patients with CM + MO not only showed fewer responses from the "correct ToM" category within the MASC for the affective dimension, but also that their errors predominantly fell into the hypo-mentalizing category. As explained earlier in this paper, hypo-mentalizing errors suggest that participants make assumptions about mental states, but these assumptions are not sufficient, unlike no-mentalizing errors. Hypo-mentalization is indeed defined as the inability or unwillingness to reflect on complex patterns of one's own mind or the minds of others. It is characterized by concrete thinking and the inability to consider mental states as motivations behind people's actions^56^. This means that CM + MO patients may be more prone to under-interpret and misunderstand intended social interaction from another human being than the EM group. The present finding is totally new for the existing literature on this topic in CM + MO. As already highlighted^30,35^, alexithymic and autism-spectrum disorders are characterized by deficits in social-emotional reciprocity, nonverbal communicative behaviors, and developing and maintaining relationships, including hypo-mentalizing impairments. Since migraine and autism share common pathophysiological changes^57–59^, it could be argued that these features may also be involved in determining similar socio-cognitive impairments as those we found in the context of the present study. Future research should explore this argument in greater depth by collecting ad-hoc measures of these aspects.

Our study is not without limitations. First, we did not collect a complete psychopathological assessment of the patients, which could be helpful in better interpreting our results, and we have not included a screening test to assess their cognitive status. However, we assessed patients’ semantic knowledge as a cognitive control variable that was previously associated with better performance in socio-cognitive abilities^46,47^. Second, an assessment of the occurrence of migraine attacks in patients with CM + MO during test administration was omitted. Therefore, we cannot rule out the potential impact of pain, which may lead to a reduction in inherent interest in the mental state of others. Third, the data collection procedure did not reflect the general migraine population because participants were recruited from a tertiary referral center. Fourth, although calculated correctly from a statistical point of view, the sample size was relatively small, which may have limited the interpretation of our results. Therefore, the transferability of these results to general practice will require confirmation on larger subgroups of patients.

Despite these limitations, the present study reports the novel application of the MASC, an ecologically valid video-based ToM task, in patients with CM + MO. If one considers that intact decoding of mental states supports everyday social interactions, it could be argued that the disruption of the ability to pick up on all nonverbal signals from others is involved in explaining the social difficulties experienced by patients with CM + MO^19,60,61^. Therefore, this impaired ability may lead these patients to respond inappropriately in social situations, eliciting negative reactions from others.

Hence, our results are doubly useful. From one side, they add a further element in the deep phenotyping of a disabling condition, which may prove to be a risk factor. From the other, they underscore the importance of optimizing the management of patients through adequate psychosocial- and social-skills interventions to be integrated into standard treatment protocols in order to prevent the evolution of EM into CM + MOH.

## Data Availability

The datasets presented in this study can be found in online repositories. The names of the repository/repositories and accession number(s) can be found below: [Zenodo; https://zenodo.org/records/8160066].

## References

[1] GBD 2015 Disease and Injury Incidence and Prevalence Collaborators, G. 2015 D. and I. I. and P. Global, regional, and national incidence, prevalence, and years lived with disability for 310 diseases and injuries, 1990–2015: A systematic analysis for the Global Burden of Disease Study 2015. *Lancet (London, England)* **388**, 1545–1602 (2016). 10.1016/S0140-6736(16)31678-6 PMC5055577 27733282

[2] Olesen, J., Headache Classification Committee of the International Headache Society (IHS), The International Classification of Headache Disorders, 3rd edition. *Cephalalgia* **38**, 1–211 (2018). 10.1177/0333102417738202 29368949

[3] Viana M (2018). Factors associated to chronic migraine with medication overuse : A cross-sectional study. Cephalalgia.

[4] Bottiroli S (2019). Negative short-term outcome of detoxification therapy in chronic migraine with medication overuse headache: Role for early life traumatic experiences and recent stressful events. Front. Neurol..

[5] Sances G (2010). Medication-overuse headache and personality: A controlled study by means of the MMPI-2: Research submission. Headache.

[6] Sances G (2013). Factors associated with a negative outcome of medication-overuse headache : A 3-year follow-up ( the ‘ CARE ’ protocol ). Cephalalgia.

[7] Galli F (2019). Personality and personality disorders in medication overuse headache : A controlled study by SWAP-200. Pain Res. Manag..

[8] Baskin SM, Lipchik GL, Smitherman TA (2006). Mood and anxiety disorders in chronic headache. Headache J. Head Face Pain.

[9] Bottiroli S (2019). Psychological, clinical, and therapeutic predictors of the outcome of detoxification in a large clinical population of medication-overuse headache: A six-month follow-up of the COMOESTAS Project. Cephalalgia.

[10] Bottiroli S (2023). Personality in chronic headache: A systematic review with meta-analysis. Pain Res. Manag..

[11] Bottiroli S, Galli F, Viana M, Sances G, Tassorelli C (2018). Traumatic experiences, stressful events, and alexithymia in chronic migraine with medication overuse. Front. Psychol..

[12] Ghiggia A (2022). Alexithymia and psychological distress in fibromyalgia and chronic migraine: A cross-sectional study. J. Psychosom. Res..

[13] Galli F (2017). Alexithymia in chronic and episodic migraine: A comparative study. J. Ment. Health.

[14] Bottiroli S (2021). Psychological predictors of negative treatment outcome with Erenumab in chronic migraine: Data from an open label long-term prospective study. J. Headache Pain.

[15] Bottiroli S (2016). Psychological factors associated with failure of detoxification treatment in chronic headache associated with medication overuse. Cephalalgia.

[16] Bottiroli S (2018). Changes in anxiety and depression symptoms associated to the outcome of MOH: A post-hoc analysis of the Comoestas Project. Cephalalgia.

[17] Andrasik F, Flor H, Turk DC (2005). An expanded view of psychological aspects in head pain: the biopsychosocial model. Neurol. Sci..

[18] Rosignoli C (2022). Applying a biopsychosocial model to migraine: Rationale and clinical implications. J. Headache Pain.

[19] Raggi A (2012). A systematic review of the psychosocial difficulties relevant to patients with migraine. J. Headache Pain.

[20] Beaudoin C, Beauchamp MH (2020). Social cognition. Handb. Clin. Neurol..

[21] Premack D, Woodruff G (1978). Does the chimpanzee have a theory of mind?. Behav. Brain Sci..

[22] Adolphs R (2003). Cognitive neuroscience of human social behaviour. Nat. Rev. Neurosci..

[23] Shamay-Tsoory SG, Aharon-Peretz J (2007). Dissociable prefrontal networks for cognitive and affective theory of mind: A lesion study. Neuropsychologia.

[24] Bottiroli S, Cavallini E, Ceccato I, Vecchi T, Lecce S (2015). Theory of Mind in aging : Comparing cognitive and affective components in the faux pas test. Arch. Gerontol. Geriatr..

[25] Bouteloup M (2021). Social and emotional cognition in patients with severe migraine consulting in a tertiary headache center: A preliminary study. Rev. Neurol. (Paris).

[26] Raimo S, D’Onofrio F, Gaita M, Costanzo A, Santangelo G (2022). Neuropsychological correlates of theory of mind in chronic migraine. Neuropsychology.

[27] Romozzi M (2022). Theory of Mind in migraine and medication-overuse headache: A cross-sectional study. Front. Neurol..

[28] Bottiroli S (2023). Social cognition in chronic migraine with medication overuse: A cross-sectional study on different aspects of mentalization and social relationships. J. Headache Pain.

[29] Song Z (2023). Psychopathic traits and theory of mind task performance: A systematic review and meta-analysis. Neurosci. Biobehav. Rev..

[30] Andreou M, Skrimpa V (2020). Theory of mind deficits and neurophysiological operations in autism spectrum disorders: A review. Brain Sci..

[31] Weng Y, Lin J, Ahorsu DK, Tsang HWH (2022). Neuropathways of theory of mind in schizophrenia: A systematic review and meta-analysis. Neurosci. Biobehav. Rev..

[32] Gillissie ES (2022). Deficits of social cognition in bipolar disorder: Systematic review and meta-analysis. Bipolar Disord..

[33] van Neerven T, Bos DJ, van Haren NE (2021). Deficiencies in Theory of Mind in patients with schizophrenia, bipolar disorder, and major depressive disorder: A systematic review of secondary literature. Neurosci. Biobehav. Rev..

[34] Crespi B, Badcock C (2008). Psychosis and autism as diametrical disorders of the social brain. Behav. Brain Sci..

[35] Dziobek I (2006). Introducing MASC: A movie for the assessment of social cognition. J. Autism Dev. Disord..

[36] Shamay-Tsoory SG (2007). Dissociation of cognitive from affective components of theory of mind in schizophrenia. Psychiatry Res..

[37] Wolkenstein L, Schönenberg M, Schirm E, Hautzinger M (2011). I can see what you feel, but I can ’ t deal with it : Impaired theory of mind in depression. J. Affect. Disord..

[38] Lee L, Harkness KL, Sabbagh MA, Jacobson JA (2005). Mental state decoding abilities in clinical depression. J. Affect. Disord..

[39] May A, Schulte LH (2016). Chronic migraine: Risk factors, mechanisms and treatment. Nat. Rev. Neurol..

[40] Baron-Cohen S, Wheelwright S, Hill J, Raste Y, Plumb I (2001). The ‘Reading the Mind in the Eyes’ test revised version: A study with normal adults, and adults with Asperger syndrome or high-functioning autism. J. Child Psychol. Psychiatry..

[41] Thurstone LL, Thurstone TG (1963). Primary Mental Abilities.

[42] von Elm E (2007). The Strengthening the Reporting of Observational Studies in Epidemiology (STROBE) statement: Guidelines for reporting observational studies. Ann. Intern. Med..

[43] Fossati A, Borroni S, Dziobek I, Fonagy P, Somma A (2018). Thinking about assessment: Further evidence of the validity of the Movie for the Assessment of Social Cognition as a measure of mentalistic abilities. Psychoanal. Psychol..

[44] Upadhyaya AK, Stanley I (1993). Hospital anxiety depression scale. Br. J. Gen. Pract. J. R. Coll. Gen. Pract..

[45] THE Whoqol GROUP (1998). Development of the World Health Organization WHOQOL-BREF quality of life assessment. Psychol. Med..

[46] Szepietowska EM, Filipiak S (2021). Interpretation of familiar metaphors and proverbs by Polish people in middle and late adulthood. Int. J. Lang. Commun. Disord..

[47] Li X (2013). Aging of theory of mind: The influence of educational level and cognitive processing. Int. J. Psychol..

[48] Richman MJ, Unoka Z (2015). Mental state decoding impairment in major depression and borderline personality disorder: Meta-analysis. Br. J. Psychiatry.

[49] Eddy CM (2019). What do you have in mind? Measures to assess mental state reasoning in neuropsychiatric populations. Front. Psychiatry.

[50] Tager-Flusberg H (2000). A componential view of theory of mind: Evidence from Williams syndrome. Cognition.

[51] Galli F (2019). Personality and personality disorders in medication-overuse headache: A controlled study by SWAP-200. Pain Res. Manag..

[52] Heaton P (2012). Measuring the effects of alexithymia on perception of emotional vocalizations in autistic spectrum disorder and typical development. Psychol. Med..

[53] Oakley BFM (2022). Alexithymia in autism: Cross-sectional and longitudinal associations with social-communication difficulties, anxiety and depression symptoms. Psychol. Med..

[54] Cook R, Brewer R, Shah P, Bird G (2013). Alexithymia, not autism, predicts poor recognition of emotional facial expressions. Psychol. Sci..

[55] Bora E, Berk M (2016). Theory of mind in major depressive disorder: A meta-analysis. J. Affect. Disord..

[56] Fonagy P (2016). Development and validation of a self-report measure of mentalizing: The reflective functioning questionnaire. PLoS One.

[57] Sahito AM (2021). Migraine and autism spectrum disorder: A shared hereditary basis [letter]. Neuropsychiatr. Dis. Treat..

[58] Vetri L (2020). Autism and migraine: An unexplored association?. Brain Sci..

[59] Sair A, Sair Y (2022). Autistic traits and obsessive-compulsive disorder in patients with migraine with or without aura. Psychiatry Behav. Sci..

[60] Cerami C (2021). High perceived isolation and reduced social support affect headache impact levels in migraine after the Covid-19 outbreak: A cross sectional survey on chronic and episodic patients. Cephalalgia.

[61] Westergaard ML, Lau CJ, Allesøe K, Andreasen AH, Jensen RH (2021). Poor social support and loneliness in chronic headache: Prevalence and effect modifiers. Cephalalgia.

